# Prof. Flint’s Prize Essay

**Published:** 1852-12

**Authors:** 


PROF. FLINT'S PRIZE ESSAY.

We have given up a large portion of the present number to the Prize Essay of Prof. Flint, assured that we could not devote it to matter more interesting to our readers. We need not bespeak for it their careful perusal; it may be be read again and agair with advantage. The author will be found to have extended the boundaries of our knowledge in reference to this important subject.

